# Fibroids and Infertility

**DOI:** 10.1007/s13669-016-0162-2

**Published:** 2016-04-25

**Authors:** P. Purohit, K. Vigneswaran

**Affiliations:** Kings College Hospital, London, UK

**Keywords:** Infertility, Fibroids, Myomectomy, Implantation, Miscarriage, Laparoscopy

## Abstract

The precise impact of fibroids, which are the most common benign gynaecological tumours in women, on reproductive function and infertility is unknown. The need to treat submucosal fibroids is widely accepted, but fibroids in other locations and sizes continue to present a clinical conundrum. This article examines the mechanisms by which fibroids affect implantation and fertility, and stratifies their impact on basis of size, location and nature. It also explores the evidence base of the available treatment modalities in specific relation to improving fertility outcomes. Traditionally, a myomectomy has been advocated to treat fibroids for the reproductive population; however, as well as evaluating the benefits of surgery including endoscopic, this article explores alternative therapies including medical and radiological interventions.

## Introduction

Fibroids are the most common uterine tumour in the reproductive age group affecting 20–50 % of these women, and hence, their relation with infertility although controversial is always a great concern to the clinician as well as the patient [1, 2••].

## Mechanisms of Infertility

Fibroids vary to a great extent in terms of their size, location and number and so does the mechanism by which they may cause infertility.

### Physical Factors

Given their size and location, it is unsurprising that simple physical impedance to the transport of sperm, egg or embryo has been proffered as a mechanism to explain the anti-fertility effects of fibroids. However, the microscopic size of the gametes and both the bilateralism and the resilience of the reproductive system suggest that this by itself is unlikely to be sole mechanism in the vast majority of cases.

### Alteration of Uterine Contractions

Uterine contractions increase in frequency in the early follicular phase from the fundus to cervix whereas in peri-ovulatory and luteal phase, their direction is reversed from the cervix to fundus [3]. Fibroids are also known to influence the contractility of the myometrium and induce a chronic inflammatory reaction, both of which may hinder implantation [4–7, 8•]. Some studies have reported increase in myometrial peristalsis in patients with intramural and submucosal fibroids when compared with healthy controls during the mid-luteal cycle phase, although there was a decrease in the peri-ovulatory phase [4–6].

Yoshino et al. using Cine mode, MRI demonstrated accelerated mid-luteal uterine peristalsis (defined as ≥2 peristaltic movements in 3 min) in the presence of intramural fibroids and achieved 40 % pregnancy rate in this population over 1 year following restoration of normal peristalsis by myomectomy [7]. Yoshino also reported another prospective study (*n* = 95) where they looked at the impact of uterine peristaltic movements due to fibroids on outcome of non-IVF fertility treatment. Thirty-four per cent women in the low-frequency group achieved pregnancy, compared with none (0 %) in the high-frequency group (*P* < 0.005) [8•].

Many underlying mechanisms have been suggested for increase in myometrial contractility like excess of cytokines, growth factors, neurotensin, neuropeptides, enkephalin and oxytocin modulators in the fibroid capsule [9].

### Cytokine Factors

Certain early pregnancy intrauterine cytokines are thought to be responsible for implantation and early embryonic development. Ben-Nagi et al. reported significant reduction in levels of certain cytokines mainly IL10 and glycodelin in the mid-luteal uterine washings of women with submucosal fibroids [10••]. Glycodelin is a progesterone-regulated glycoprotein secreted into uterine luminal cavity by secretory/decidualized endometrial glands and has properties like promoting angiogenesis and suppressing natural killer (NK) cells.

### Genetic

Endometrial HOXA10, HOXA11 and BTEB1 gene expression has been shown to modulate endometrial receptivity. The reduction or absence of HOXA10 in the uterine endometrium leads to infertility due to the inability of the embryo to implant [11•]. Rackow et al. demonstrated a significant reduction in concentration of these genes during follicular phase in infertile women with submucosal fibroids (FIGO L0 to L2). Interestingly, the reduction was present throughout the uterine cavity and not just in the endometrium overlying the fibroid. There was no significant decrease for intramural (IM) fibroids, although a trend to lower levels was noted [5]. On the other hand, Matsusaki et al. was able to demonstrate a significant decrease in HOXA10 concentrations during luteal phase in infertile women with intramural fibroids compared to healthy patient controls [12].

The downregulation of endometrial HOXA 1 gene expression results in defective decidualization possibly mediated via secretion of transforming growth factor beta3 (TGF-β3) [13]. Alizadeh reported increase in endometrial HOXA1 gene expression following myomectomy [14].

### Alterations in the Endo-myometrial Junctional (EMJ) Zone

The EMJ which represents the inner 1/3rd of the myometrium abutting the endometrium contributes macrophages and uterine natural killer (uNK) cells which are essential for the process of endometrial decidualization in the mid-luteal window of implantation. In women with uterine fibroids, Kitaya et al. found significant reduction in concentrations of both macrophages and uNK cells in the EMJ, thus, negatively affecting implantation [15]. Also, it is possible that the presence of intramural or submucosal fibroids physically disrupts the EMJ and alters the steroid receptors, leading to implantation failure [16].

## Infertility and Reproductive Outcomes

The evidence base in relation to fibroids and infertility is complex, with an overrepresentation of observational data and a lack of well-designed controlled trials. Moreover, the heterogeneity in patient populations and fibroid disease and multifactorial aetiology of infertility mean that it is often difficult to plan and successfully execute large scale multi-centre randomised controlled trials.

So far, we have explored biological plausibility by which fibroids may cause infertility. In this section, we will explore the evidence base for harm and treatment benefit.

### Evidence of Harm: Does Presence of Uterine Fibroids Reduce Implantation Rates?

It is generally accepted that submucous fibroids have a negative impact on fertility and early pregnancy by the virtue of their involvement in the endometrial cavity. A systematic review by Pritts et al. concluded that submucosal fibroids (FIGO L0 to L2) which cause distortion of the uterine cavity resulted in the decreased rates of clinical pregnancy, implantation and ongoing pregnancy/live birth, as well as an increased rate of spontaneous miscarriage [17••].

In contrast to this, there is a considerable controversy regarding fibroids that do not cause distortion of the uterine cavity. The review by Pritts et al. found that women with fibroids with no submucosal involvement, i.e. pure intramural fibroids (FIGO L3 to L4), had decreased rates of implantation and ongoing pregnancy/live birth, and an increased rate of spontaneous miscarriage when compared with controls without fibroids. One weakness of Pritts’ review is that most of the studies included did not use a formal means such as hysteroscopy or saline sonography to exclude the involvement of the uterine cavity, i.e. there may be an ascertainment bias and overestimation of effect size in that some of the cases deemed as intramural and may have an undiagnosed submucosal component [17••]. What is clear from the review is that there was no evidence to suggest that subserosal (FIGO L5 to L7) fibroids decreased any measure of fertility.

A synthesis of available evidence shows a 21 % reduction in live birth rates following in vitro fertilization (IVF) in women with non-cavity distorting intramural fibroids, when compared with non-fibroid controls [18••]. The group whilst acknowledging the inherent weakness of the review owing to the heterogeneity of patient populations highlighted the relatively lower chance of achieving a live birth, when compared with clinical pregnancy rate, and attributed this to increased rates of miscarriage and premature birth [18••].

A major confounding factor in infertility success is access to health care. There is a strong residual effect in that women who receive treatment early are most likely to have successful outcomes, whereas those who suffer long duration of infertility are the residuals whose prognosis is worse irrespective of treatment.

There is plethora of evidence that, Afro-Caribbean women, in whom fibroids are more common and more severe, have poor access to health care compared to Caucasian women, and therefore under-represented in ART databases [19]. Feinberg et al. examined the disparity in outcomes of the first non-donor IVF cycles between African-American patients and Caucasian patients, in the Department of Defence population, which is an equal access to care setting [20]. Fibroids were approximately three times as common in African-American as opposed to Caucasian women (30.8 versus 10.7 %). Women were offered routine saline sonography prior to IVF, and those with fibroids larger than 3 cm or submucosal component offered surgery. Although, the study could neither ascertain the staging of fibroids at baseline scan nor the proportion of patients who underwent surgery, African-American women were found to have statistically significant higher rates of miscarriage, when compared to Caucasian patients and fibroids were thought to be a contributing factor for this variation [20]. The reproductive outcomes between the two groups were similar when adjusted for fibroids. In both groups of women, the presence of fibroids at baseline scan reduced IVF implantation and live birth rates by 18 % (95 % CI 2–31 %) and 27 % (95 % CI 4–44 %), respectively [20].

### Evidence of Treatment Benefit: Does Treatment of Uterine Fibroids (For Example Myomectomy) Improve Fertility Rates and Outcomes?

There are many case series reporting the benefits of myomectomy. For example, Babaknia et al. in 1978 reported 38 % term pregnancy rate following myomectomy in 34 women with otherwise unexplained infertility [21•].

Casini et al. reported the only RCT published to date, in the group of women with fibroids but otherwise unexplained infertility [22••]. All women except for those whose fibroids were purely subserous, i.e. no intramural component (*n* = 11), were included and randomised (total randomised *n* = 170) to undergo myomectomy or not and spontaneous conception rates observed over 12 months following surgery. All women undergoing myomectomy, which was carried out either hysteroscopically or by laparotomy, reported increased pregnancy rates irrespective of baseline fibroid staging [22••]. Statistically, significant increase was, however, only observed in women with submucosal fibroid [pregnancy rates myomectomy versus no myomectomy; submucosal group = 43.3 versus 27.2 %; intramural with submucosal component = 40 versus 15 %; all submucosal = 21/52 (40.4) versus 9/42 (21.4 %)]. The pregnancy rates of the 11 women excluded on basis of pure subserous staging was 63.6 % [22••].

Whilst there are many non-randomised controlled trials in published literature, their common fallacy is the choice of inappropriate controls, i.e. to give a valid answer, both the treatment and control arm should suffer disease in question of uterine fibroids. It is not correct to compare women who undergo myomectomy with infertile controls who do not have fibroids at all. In absence of appropriate controls, evidence from studies where patients serve as their own internal controls, i.e. a before and after effect, is acceptable, although not without its own methodological problems.

A review of literature reveals only one such adequately controlled trial, albeit non-randomised, which investigated the treatment effect of myomectomy prior to IVF [23]. All the patients selected had between one and five fibroids, with one measuring at least 5 cm and all without a submucosal component. The study established the beneficial effects of pre-IVF myomectomy, as shown by the 25 % delivery rate in the myomectomy group, when compared to the 12 % delivery rate in the no surgery group.

A Cochrane review of three RCT’s [22••, 24, 25] concluded that there is insufficient evidence to recommend a myomectomy for the purpose of improving fertility outcomes in the case of intramural or subserosal fibroids [26••].

In summary, the published literature make clear divisions between the location of fibroids and the benefit of myomectomy on reproductive outcomes, both in terms of spontaneous pregnancies as well as IVF outcomes. The consensus based on clinical experience would appear to imply very little causation linking subserosal fibroids and infertility. Therefore, unless there were other indications, a myomectomy to remove subserosal fibroids for infertility is not evidence based. Submucosal fibroids, on the other hand, are shown to lower fertility rates and studies have demonstrated by removing such fibroids; there is an improvement in both conception and live birth rates.

With regard to intramural fibroids, both the evidence and consensus for myomectomy, purely for infertility, is weak. Given the risk of significant morbidity of surgery including that of postoperative adhesion formation, particularly those performed through posterior uterine incisions, [27] further research is outstanding and cases have to be managed on an individual basis.

## A Pragmatic Approach to Management

Attribution is the exercise of determining a causal association between a finding and a symptom, i.e. the exercise of establishing causation. In vast majority of cases, it is difficult or even impossible to ascertain causation with absolute certainty, and therefore, the attribution exercise should also include a means of determining the strength/likelihood of the causal association, so a treatment effect can be estimated.

Accurate fibroid mapping, i.e. description of size, location and nature of fibroids, using ultrasound scan is a critical step in such an assessment. Saline infusion sonography (SIS) can be used to rule out submucosal involvement, and MRI reserved for complex cases or to differentiate from adenomyosis.

**Fig. 1:**
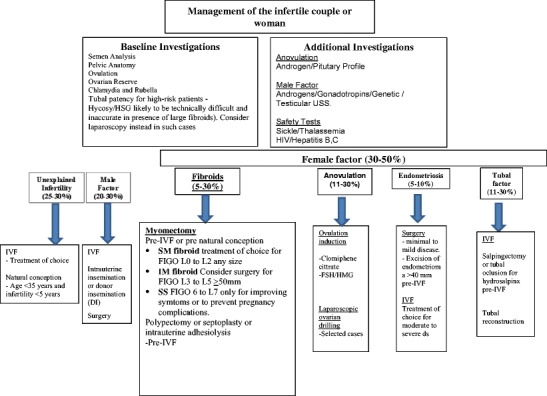
Overview of investigations and treatment of infertility (adapted with permission from Yalandu and Narvekar, Wiley publishing) [28]

All patients should complete preliminary investigations to ascertain causation of infertility; the important domains include assessment of ovarian reserve and ovulation, as well as seminal fluid analysis. Tubal patency tests are invasive, and in the presence of fibroids are inaccurate [28]. Accordingly, patency should be assessed opportunistically at time of myomectomy, or if indicated, by HSG or HyCoSy as appropriate. An overview of the investigations and treatment of infertility is outlined in Fig. 1 (adapted with permission from WILEY-TOG article) [28].

The two critical factors which help assess the need for treatment are (a) the absence or presence of other causal aetiologies and (b) the overall chance of conception, whether natural or otherwise. For example, if fibroids are a part of a multi-factorial aetiology, it is difficult to determine which aetiology is most causative in infertility. On the other hand, if the aetiology is otherwise unexplained, then it is appropriate to consider treatment either surgical or medical for the fibroids. The overall chance of conception is also an important factor in decision making. For example, the removal of submucous or large intramural fibroids is likely to be successful in a woman age <40 with otherwise unexplained infertility, as opposed to a woman age 40 or more with low/poor ovarian reserve, where myomectomy irrespective of size and location of fibroids is unlikely to be of major benefit.

## Surgical Treatments

### Hysteroscopic Myomectomy

If the fibroids are predominately located within the cavity (FIGO L0, L1), hysteroscopic myomectomy would appear to help restore cavity dimensions and subsequently improve fertility outcomes. The risk of endometrial damage and intrauterine adhesions, and its subsequent effect on conception and pregnancy outcomes, has to be discussed with patient during pre-operative counselling.

Intrauterine adhesions have been reported to occur in up to 7.5 % of hysteroscopic myomectomies [29]. Valle et al. showed that increasing severity of intrauterine adhesions correlated with corresponding decrease in reproductive outcomes. This ranged from a term pregnancy rate of 81.3 % in patients with mild disease to 31.9 % in patients with severe disease [29].

FIGO L2 fibroids, whereby less than 50 % of the fibroid is located with the cavity, are more difficult to resect and may require a two-stage procedure, especially if larger than 3 cm. Camanni et al. demonstrated that hysteroscopic approach is suitable for fibroids measuring up to 5 cm in diameter [30]. One has to exercise extreme caution in assessing what is feasible technically and what is best for the management of patient’s symptoms. Whilst it is perfectly reasonable to perform resection of large L2 fibroids, albeit in multiple procedures, for the management of severe menstrual symptoms, the risk of endometrial damage and adhesions may negate any fertility benefits. As such, for infertility, it may be prudent to remove such fibroids by laparoscopy, although it does increase the risks associated with a full thickness myometrial incision such as uterine rupture in the future pregnancy and labour.

### Laparoscopic Versus Laparotomy

All fibroids FIGO L3 and above (and large L2 as outlined above) are best removed by laparoscopy or laparotomy. The improvement in reproductive outcomes appears to be similar by both the approaches. Combined data of 267 women from two RCTs comparing laparoscopic myomectomy and abdominal myomectomy demonstrated similar reproductive outcomes in both groups [24].

In the first study of 131 patients undergoing myomectomy for infertility and at least 1 fibroid > 5 cm, pregnancy rates were similar in the laparoscopy and laparotomy groups (53.6 versus 55.9 %).Febrile morbidity was reduced in the laparoscopy group (26.2 versus 12.1 %), when compared with laparotomy as well as a smaller mean drop in haemoglobin and a shorter inpatient stay [24]. In the second study involving 132 women with fibroids, whilst cumulative outcomes within the first 12 months following surgery were similar (cumulative pregnancy rate 52.9 versus 38.2 %), the per cycle outcomes such as pregnancy rate per cycle (6.5 versus 3.9 %) and time to the first pregnancy (WMD = 1 month) were significantly higher in the laparoscopic compared to the laparotomy group [25].

### Medical Treatments

Medical treatments such as combined oral contraceptive pill (COCPs), progesterone only-pill (POP) and levonorgestrel intrauterine system (LNG-IUS), whilst useful in managing menstrual and pain symptoms, are contraceptive and therefore not applicable to the infertile women. Other medical treatments such as mefenamic and tranexamic acid can be safely prescribed [31].

Ulipristal acetate (UPA), a selective progesterone receptor modulator is now approved and licensed for the medical treatment of uterine fibroids in many countries. UPA has been shown to improve menstrual symptoms and lead to regression in fibroid size [32]. The regressive effects are maintained for 6 months, primarily because the compound increases apoptosis of leiomyoma cells [33•]. This phenomenon has allowed for intermittent dosing, and UPA is now licensed accordingly [34]. The maximum duration of therapy is 3 months, and the recommended interval between therapies has to be a minimum of two washout-menstrual cycles, which also allows for any endometrial changes, the so-called PAEC to revert to normal.

UPA is marketed in strengths of up to 100 mg for emergency contraception [35]; however, contraceptive effects of a daily 5 mg dose are unknown, and therefore, patients should be advised to use alternate contraception such as condoms during therapy in order to avoid any teratogenicity. Any conception benefits of UPA, resulting from fibroid regression, have to be evaluated following end of therapy or in the washout cycles if prescribed intermittent dosing.

Luyckx et al. reported the first series of 18 such pregnancies in 52 women participating from a single centre in Pearl II and Pearl III studies. Thirty-seven women were treated with one-off 3-month UPA therapy (Pearl II, Pearl III) and 15 with intermittent therapy lasting a total of 6 to 12 months (Pearl III extension). Of the 21 women who wished to conceive after completion of UPA therapy, 19 underwent myomectomy, and 2 were not. Seventy-one per cent (15/71) women conceived for a total of 18 times, 12 of which were spontaneous and a further 6 achieved with IVF [36]. There were a total of 13 live births (1 twin) and 6 miscarriages. The two women, who did not undergo myomectomy, had a total of three pregnancies between them, but only one live birth [36].

Whilst the data shows feasibility and safety of conception after UPA therapy, it also highlights the high miscarriage rate in the presence of fibroids despite reduction in size (2/3 versus 4/15 conceptions in women who did not undergo myomectomy versus those who did) and therefore the superiority of myomectomy over reductive therapies.

## Uterine Artery Embolization

Uterine artery embolization was first described in 1995 by Ravina as an alternative radiological treatment option for women with large fibroids no longer desiring their fertility [37]. MRI imaging shows a transient ischemia within the body of the uterus and the endometrium typically lasting for up to 72 after the uterine artery embolization (UAE) procedure. This ischemic change is intended to be irreversible within fibroid tissue only, and temporary within healthy uterine muscle and endometrium, but nevertheless, raises concerns regarding its effect on whole uterine and endometrial function. Also, the uterine and ovarian artery has been shown to anastomose on angiography, in at least one side in approximately 46 % of women. Therefore, inadvertent embolization of ovarian tissue may result in premature ovarian insufficiency and failure especially in older women or those with low baseline ovarian reserve. Reassuringly, the reported incidence of amenorrhea in the under 40 age group is less than 1 %.

Mara et al. conducted an RCT evaluating UAE versus abdominal myomectomy in an infertile population [38]. The pregnancy rates were 50 and 78 % in the UAE and myomectomy arms, respectively. They recruited young patients below age 35 [mean age 32 (SD ±4.1) years] which may explain the high conception rates overall. Also, the latency period, i.e. time to conception was longer for UAE (mean = 18 months) compared with myomectomy (mean = 13 months). The re-intervention rates were higher (19 out of 58) in the UAE arm, as has been observed in other studies [39].

Following a systematic review of the published literature, Homer et al. reported a 35.2 % rate of miscarriage in UAE conceptions as compared to 16.5 %in fibroid-containing pregnancies (odds ratio [OR] 2.8; 95 % confidence interval [CI] 2.0–3.8) matched for age and fibroid location [40]. There was a higher incidence of caesarean section and PPH in the UAE pregnancies, whereas rates for preterm delivery and malpresentation were similar in the two groups.

Given the current evidence base, UAE is not a treatment of first choice for women with infertility or those desirous of future fertility. Instead, it is to be reserved for poor surgical candidates.

## Magnetic Resonance-Guided Focused Ultrasound Surgery

Another alternative treatment modality which has demonstrated encouraging preliminary results is the use of magnetic resonance-guided focused ultrasound surgery (MRgFUS). This treatment involves the application of MRI-directed beams of ultrasound capable of heating an area of fibroid tissue to up to 70 °C and causing destruction through coagulative necrosis.

Rabinovici et al. reviewed all pregnancies reported to the FDA MAUDE (manufacturer and user facility device experience) database following MRgFUS. In total, 54 pregnancies were reported in 51 women with a mean age at MRgFUS of 37.2 years and mean time to conception of 8 months [41, 42]. The miscarriage rate was 28 %. The preliminary experience is encouraging, with a high rate of delivered and ongoing pregnancies.

## Conclusion

The evidence regarding effect of fibroids on infertility and reproductive outcomes is weak and mostly inconclusive. In infertile women, appropriate evaluation and classification of fibroids, particularly those involving or suspected to be involving the endometrial cavity is essential. Submucosal fibroids (FIGO L0-L2) should be treated hysteroscopically (or laparoscopic for large L2) to improve conception rates. The management of intramural fibroids should be individualised on a case to case basis, whereas subserosal fibroid are unlikely to have any major impact on fertility. Conservative treatment measures (Medical, UAE and MrgRUS) should not be routinely offered to women who wish to maintain or improve their fertility due to lack of data on their safety and effectiveness.
